# Reducing Waiting Times from Referral to Assessment in a Memory Service: A Quality Improvement Project

**DOI:** 10.1192/bjo.2026.11506

**Published:** 2026-06-30

**Authors:** Muge Ulusoy Altinoklu, Emma Reames, Marta Rato, Tara West, Alan Smith

**Affiliations:** Central and North West London HS Foundation Trust, London, United Kingdom

## Abstract

**Aims:**

Dementia affects approximately 850,000 people in the United Kingdom, and early diagnosis is a key priority outlined in National Institute for Health and Care Excellence (NICE) guidelines (1, 2). Increasing referral rates and service capacity pressures have resulted in prolonged waiting times for memory services, adversely affecting patient safety, clinical outcomes, and carer distress.

The Kensington & Chelsea and Westminster (KCW) Memory Service receives approximately 30–40 referrals per week. This Quality Improvement Project (QIP) aimed to improve quality and patient safety by reducing the time from referral to initial memory assessment within the KCW Memory Service.

**Methods:**

A multidisciplinary team undertook a QIP using iterative Plan–Do–Study–Act (PDSA) cycles. Primary drivers were identified,including: (I) number of assessments completed per week, (II) delays awaiting brain imaging, (III) reporting times, (IV) multidisciplinary team meeting processes, and (V) triage. Baseline measures included waiting time from referral to assessment and monthly dementia diagnosis rates. Outcome measures were reviewed monthly, impact of changes over time, care quality and patient safety were maintained. Service user involvement by feedback and questionnaire was incorporated to support evaluation and inform ongoing improvement.

**Results:**

The average time from referral to assessment was reduced by more than one third, from over 120 days in March 2025 to 72 days by January 2026. Over the same period,monthly dementia diagnosis rates increased from 4 diagnoses in February 2025 to approximately 50 diagnoses per month following implementation of the interventions. The number of individuals waiting longer than 18 weeks for assessment decreased from 180 in March 2025 to 55, while the number of referrals awaiting triage reduced from 199 in April 2025 to fewer than 10 currently.

During this period, the service also received positive feedback from service users, reflecting improved experiences related to timeliness and communication.

**Conclusion:**

This QIP demonstrates that structured, data-driven service redesign can significantly reduce waiting times and improve diagnostic capacity without compromising care quality. These findings indicate a meaningful and sustained improvement in service performance. Reduced waiting times has important secondary benefits for patient safety, including earlier identification of clinical risk and reduced distress for patients and carers. Ongoing incorporation of service user involvement supports the acceptability and sustainability of the improvements. The approach may be transferable to other memory services facing similar demand and capacity challenges.

References:

1. NHS England. (n.d.). Dementia. NHS England. https://www.england.nhs.uk/north-west/north-west-coast-strategic-clinical-networks/our-networks/adult-children-and-young-peoples-mental-health-and-dementia/dementia/

2. Dementia: assessment, management and support for people living with dementia and their carers, NICE guideline, Published: 20 June 2018

